# Low pH and Anionic Lipid-dependent Fusion of Uukuniemi Phlebovirus to Liposomes*

**DOI:** 10.1074/jbc.M115.691113

**Published:** 2016-01-25

**Authors:** David Bitto, Steinar Halldorsson, Alessandro Caputo, Juha T. Huiskonen

**Affiliations:** From the Division of Structural Biology, Wellcome Trust Centre for Human Genetics, Roosevelt Drive, University of Oxford, Oxford OX3 7BN, United Kingdom

**Keywords:** electron tomography, membrane, membrane fusion, virus entry, virus structure, bis(monoacylglycero)phosphate, bunyavirus, phlebovirus

## Abstract

Many phleboviruses (family *Bunyaviridae*) are emerging as medically important viruses. These viruses enter target cells by endocytosis and low pH-dependent membrane fusion in late endosomes. However, the necessary and sufficient factors for fusion have not been fully characterized. We have studied the minimal fusion requirements of a prototypic phlebovirus, Uukuniemi virus, in an *in vitro* virus-liposome assay. We show that efficient lipid mixing between viral and liposome membranes requires close to physiological temperatures and phospholipids with negatively charged headgroups, such as the late endosomal phospholipid bis(monoacylglycero)phosphate. We further demonstrate that bis(monoacylglycero)phosphate increases Uukuniemi virus fusion beyond the lipid mixing stage. By using electron cryotomography of viral particles in the presence or absence of liposomes, we observed that the conformation of phlebovirus glycoprotein capsomers changes from the native conformation toward a more elongated conformation at a fusion permissive pH. Our results suggest a rationale for phlebovirus entry in late endosomes.

## Introduction

The family *Bunyaviridae* comprises the largest group of viruses and is divided into five genera (*Phlebovirus, Orthobunyavirus, Nairovirus, Hantavirus,* and *Tospovirus*). Members of this family are enveloped and harbor a negative-sense, segmented single-stranded RNA genome (1). Members of the genus *Hantavirus* are transmitted to humans from rodents, whereas members of the genera *Phlebovirus*, *Orthobunyavirus*, and *Nairovirus* are zoonotic arboviruses; they are transmitted to humans from their animal reservoirs via arthropod vectors, such as mosquitoes or ticks (2). Tospoviruses, which infect plants, are also transmitted by arthropods, such as thrips (3).

Notable examples of highly pathogenic bunyaviruses include Crimean-Congo hemorrhagic fever virus (*Nairovirus*) (4) and members of the *Phlebovirus* genus, such as Rift Valley fever virus (5), and severe fever with thrombocytopenia syndrome virus (6, 7). The former, as well as the recently emerged Schmallenberg virus (*Orthobunyavirus*), have been associated with significant loss of livestock (5, 8). Despite the medical and agricultural relevance of bunyaviruses, we are only starting to unravel how they enter their host cells. Understanding virus entry in detail can be of paramount importance in designing antiviral therapies, none of which are currently available to specifically treat bunyaviral diseases.

Many enveloped viruses enter the cell by endocytosis, as originally demonstrated for Semliki Forest virus (SFV)^3^ (*Alphavirus*, *Togaviridae*) in 1980 (9, 10). Within the family *Bunyaviridae*, this process is best understood for phleboviruses. A C-type lectin, dendritic cell-specific intercellular adhesion molecule-3-grabbing non-integrin has been shown to mediate entry of phleboviruses into a number of cell lines, including dendritic cells (11). Endocytic routes of phlebovirus infection have been shown to be largely clathrin-independent (12) and caveolin-dependent (13). After endocytosis, phleboviruses are transported to early endosomes where they detach from their receptor and continue to late endosomes (LEs) (11), from which viral penetration into the cytosol is thought to take place (12), presumably via membrane fusion catalyzed by the viral fusion glycoprotein G_C._ The recently solved crystal structure of Rift Valley fever virus (RVFV) G_C_ revealed a classical class II fusion protein architecture (14), observed earlier in alphaviruses (15) and flaviviruses (16). G_C_, together with the glycoprotein G_N_, forms laterally connected, ring-shaped assemblies filling the virion surface (17, 18). Structural similarities in the fusion proteins suggests that the membrane fusion mechanism of phleboviruses may be similar to that of other late penetrating viruses harboring class II fusion proteins, such as dengue virus (*Flavivirus*, *Flaviviridae*) (19, 20). However, the phlebovirus fusion mechanism has remained elusive.

We used fluorescence spectroscopy and electron cryotomography to study the fusion mechanism of the prototypic phlebovirus, Uukuniemi virus (UUKV), in a virus-liposome model system. The types of phospholipid species, in addition to pH and temperature, affected membrane fusion. The presence of the late endosomal phospholipid bis(monoacylglycero)phosphate (BMP) (21) in the liposomes facilitated viral fusion below pH 5.6 and at physiological temperatures. Electron cryotomography of virions and virus-liposome complexes provided direct visualization of different stages of fusion, ranging from the prefusion state of the viral glycoprotein capsomers to a more extended conformation mediating binding to the target membrane and putative fusion of the two membranes. Taken together, our data provide a rationale for phlebovirus fusion in late endosomes (12), which may not only be facilitated by late endosomal pH, but possibly also by the compartment-specific phospholipid BMP.

## Experimental Procedures

### 

#### 

##### Cells and Viruses

Baby hamster kidney (BHK)-21 cells were provided by Anna Överby (Umeå, Sweden) and grown in Glasgow minimal essential medium (Life Technologies, Thermo Fisher Scientific, Waltham, MA), 5% FBS (PAA Labs, USA), 10% tryptose phosphate broth (Sigma), and 20 mm HEPES (Life Technologies, Thermo Fisher Scientific) at 37 °C in the presence of 5% CO_2_. UUKV was produced in BHK-21 cells and purified as previously described (12). Briefly, viral supernatants were cleared from cell debris by low speed centrifugation, and the virus particles concentrated by ultracentrifugation of the clarified supernatant through a 20% sucrose cushion at 25,000 rpm (SW32 rotor; Beckman Coulter, Pasadena, CA). Viral pellets were allowed to dissolve overnight in neutral buffer at 4 °C and purified over a linear sucrose gradient. For electron cryotomography of virus-liposome fusion complexes, sucrose gradient-purified UUKV was diluted 10-fold in sucrose-free neutral pH buffer to reduce sucrose concentration, and pelleted again at 4 °C for 1 h at 35,000 rpm (TLS-55 rotor; Beckman Coulter, Pasadena, CA). The virus pellet was resuspended in a small remainder of supernatant. For electron cryotomography of isolated virions, pelleting of the virus was avoided. Instead, clarified supernatants were concentrated using centrifugation at 3,000 × *g* in an ultrafiltration device with a 30-kDa cutoff (Vivaspin15R; Sartorius AG, Göttingen, Germany), followed by purification over a linear gradient (8–50% (w/v) Nycodenz; Progen Biotechnik, Heidelberg, Germany).

Metabolic labeling of UUKV with 1-pyrenehexadecanoic acid (Life Technologies) was performed similarly as described for other viruses (22–24). Briefly, cells were incubated for 48 h in 15 μg/ml of 1-pyrenehexadecanoic acid in growth media prior to infection. Cells had to be of low passage number to minimize the cytopathic effect caused by the fluorescent probe. The probe was washed away with serum-free media and cells were infected as described above, in the absence of the fluorescent probe. Pyrene-labeled UUKV was concentrated by pelleting and subsequently purified over a sucrose gradient by ultracentrifugation, as indicated above. Virus specific infectivity was estimated by focus-forming unit (FFU) titration (12) and non-reducing SDS-PAGE of virus preparations, using a bovine serum albumin (Thermo Fisher Scientific) standard.

SFV production and pyrene labeling was done essentially as described here for UUKV. Virus production time was 20–24 h, no sucrose cushion was used in ultracentrifugation and a standard plaque-forming unit (pfu) assay using crystal violet stain (Sigma) was used to determine virus infectivity. As an alternative way to quantify relative concentrations of pyrene-labeled virus stocks, fluorescence emission levels were assessed in the presence or absence of 0.2% Triton X-100 by recording emission spectra using a spectrophotometer (Cary Eclipse; Agilent Technologies, Santa Clara, CA). Filters used were 250–395 nm for excitation and 360–1100 nm for emission, using slit-widths of either 5 or 10 nm.

##### Lipids and Liposomes

1,2-Dioleoyl-*sn*-glycero-3-phosphocholine (DOPC), 1,2-dioleoyl-*sn*-glycero-3-phosphoethanolamine (DOPE), BMP (*S*,*R*-isomer), cholesterol (ovine wool, >98%), sphingomyelin (brain, porcine), 1,2-dioleoyl-*sn*-glycero-3-phospho-(1′-rac-glycerol) (DOPG), 1,2-dioleoyl-*sn*-glycero-3-phospho-l-serine (DOPS), and l-α-phosphatidic acid (PA; egg, chicken) were purchased from Instruchemie BV (Delfzijl, Netherlands) and 1-hexadecanoyl-2-(1-pyrene-decanoyl)-*sn*-glycero-3-phosphocholine (PC-pyr) was purchased from Thermo Fisher Scientific. All lipids were dissolved in chloroform, except PC-pyr, which was dissolved in ethanol. Dissolved lipids were mixed together in the required molar proportions (Table 1) and the solvent was evaporated in an argon stream. The dried lipids were hydrated to a final concentration of 2 mm total lipid, using 6 mm succinate, 22 mm phosphate, 22 mm glycine (SPG buffer), pH 7.5, including 100 mm NaCl (SPG50N100), by vortexing and thoroughly pipetting up and down. Liposomes containing 1-hexadecanoyl-2-(1-pyrenedecanoyl)-*sn*-glycero-3-phosphocholine were assessed for pyrene fluorescence by fluorometry, as described above for pyrene-labeled virions. For content mixing assays, liposomes were resuspended in SPG buffer including 75 mm NaCl and 50 mm sulforhodamine B (SRB; Life Technologies). For production of SRB-containing liposomes, lipid hydration was done by shaking the lipids in buffer for 45–60 min at room temperature, followed by thorough resuspension, and performing 10 freeze-thaw cycles, alternating between dry ice and 37 °C. Liposomes were prepared by extruding hydrated lipids 21 times through polycarbonate membranes (Whatman; GE Healthcare Life Sciences, Buckinghamshire, UK) of 100 nm pore size at room temperature, using a mini-extruder (Avanti Polar Lipids, Alabaster, AL). Lipid mixtures containing sphingomyelin were extruded at 37 °C to account for elevated transition temperatures. Excess dye in SRB-labeled liposomes was removed by passing the preparation through a PD Miditrap G-25 column (GE Healthcare Life Sciences). The first 10 drops containing the liposomes were collected. Liposome size homogeneity within samples was confirmed by dynamic light scattering.

##### Lipid Mixing Assays

Lipid mixing assays were done similarly as previously described for other pyrene-labeled viruses (22–25). UUKV-pyr (2.4–6.9 × 10^7^ FFU) or SFV-pyr (1.2–10 × 10^8^ PFU) at similar excited dimer fluorescence levels were mixed with an excess of unlabeled liposomes (100 μl of 2 mm input lipid, assuming insignificant loss of lipids during rehydration) in SPG buffer and 100 mm NaCl (SPG50N100) to a final volume of 1 ml, leading to a maximal sucrose concentration of ∼1.2% (w/v) (assuming a maximum sucrose concentration of 40% in viral stocks) and a total lipid concentration of 200 μm. Mixtures were preincubated at 37 °C for 10 min in a quartz cuvette (Hellma Analytics, Müllheim, Germany), after which a pre-titrated amount of 1 m HCl was added to reach the intended low pH. Pyrene excimer fluorescence was measured at 37 °C for up to 10 min after acidification. Lipid mixing at lower temperatures, as well as in presence of pyrene-labeled liposomes, was performed in the same way. Final pH was verified using a pH microelectrode (Mettler Toledo, Zurich, Switzerland). Total pyrene excimer dilution was induced by addition of a final concentration of 0.2% (w/v) Triton X-100 (Sigma). Measurements were done using a spectrophotometer (Cary Eclipse; Agilent Technologies, Santa Clara, CA) at 345 nm excitation and 475 nm emission wavelengths with 10-nm slit width. Filters used were 250–395 nm for excitation and 430–1100 nm for emission. To calculate lipid mixing indices, the pyrene excimer fluorescence emission decrease was scaled between an average intensity at neutral pH (defined as 0% lipid mixing), shortly before acidification, and an average after the addition of Triton X-100 (defined as 100% lipid mixing).

##### Content Release Assays

In content release assays, SRB-labeled liposomes, equivalent to a total de-quenched fluorescence of 600 to 800 arbitrary units (using 700 V photomultiplier voltage), were mixed with UUKV-pyr. The virions were present in similar amounts as used in lipid mixing assays above, but the liposomes were not assumed to be in excess anymore. This experimental setup was chosen to allow simultaneous detection of both UUKV-pyr (excitation, 345 nm; emission, 475 nm, 5 nm slit width) and SRB-labeled liposomes (excitation, 565; emission, 586 nm, 5-nm slit width). Excitation filter 335–620 nm and emission filter 430–1100 nm were used. The acidification, complete de-quenching, and quantification were done essentially as described above for the lipid mixing assay. To calculate the content release index, the SRB emission was scaled between emission at neutral pH (defined as 0% content release) and emission in the presence of 0.2% Triton X-100 (defined as 100% content release).

##### Cell-Cell Fusion Assays

Cell-cell fusion mediated by UUKV or SFV particles at the cell surface was performed similarly as described earlier (12, 20), with the following modifications. Briefly, BHK-21 cells were washed once in serum-free binding medium (serum-free BHK-21 growth media in presence of 0.2% (w/v) cell culture grade BSA; Life Technologies) and then incubated for 5 min in the presence of 5 μm BMP at 4 °C. After removal of excess BMP, UUKV (multiplicity of infection ∼100) or SFV (multiplicity of infection ∼70) was added in serum-free binding medium and allowed to bind to cells for 60 min at 4 °C. To induce fusion, virus/cell mixtures were treated with binding medium supplemented with 30 mm citrate (final pH 5.0) for 5 min at 37 °C. This was followed by a 30-min incubation at 37 °C in normal binding medium to facilitate full syncytia formation. In some control experiments, BMP was added to binding medium only at this stage. After fixation in 4% (w/v) formaldehyde in PBS, cell nuclei were stained with Hoechst (8 μg/ml; Life Technologies, Thermo Fisher Scientific) and membranes with Cell Mask Orange (2 μg/ml; Life Technologies, Thermo Fisher Scientific) and imaged in PBS. Fluorescence microscopy was performed at room temperature with a Zeiss Observer Z1 spinning disk confocal microscope and a Zeiss ×40 C-Apochromat NA 1.2 water-immersion objective. Images were acquired from three different experimental replicates for each condition using the Zeiss ZEN Blue software and fusion indices F were calculated as previously described (12), using the following formula: *F* = [1 − (*C*/*N*)], where *C* indicates the number of cells and *N* the number of nuclei per field of view (a total of 31–46 fields of view per condition).

##### Electron Cryomicroscopy and Tomography

An aliquot (3–4 μl) of purified virions or virus-liposome complexes was pipetted onto a glow-discharged holey carbon grid (C-flat; Protochips, Raleigh, NC) and 1 μl of BSA-coated 10-nm gold beads (Aurion; Wageningen, The Netherlands), used as fiducial markers, was added. For electron cryotomography of isolated virions, the grids were washed against pH 7.5 buffer to remove residual Nycodenz (26). For virus-liposome complexes, unlabeled, low sucrose-containing UUKV (∼0.1 μm glycoprotein end concentration) was first mixed with an equal volume of BMP-liposomes at 1 mm end concentration and incubated at 37 °C for 10 min to allow possible binding. The grids were washed against 2 ml of SPG50N100 adjusted to pH 5.0 or 7.5 (control) at 37 °C for 20 s. Each grid was blotted in a humidified chamber (∼70–80% relative humidity) manually from the side opposite of the sample with filter paper to remove excess sample, followed by immediate vitrification in liquid ethane-propane using a vitrification apparatus (CP3; Gatan, Pleasanton, CA). Single-axis tomographic tilt series were collected at 3° increments from −60° to 60° using SerialEM (27) and an electron microscope operated at 300 kV (Tecnai F30 “Polara”; FEI, Hillsboro, OR) in low dose mode. Inelastically scattered electrons were filtered in zero energy-loss mode using an energy filter (Quantum; Gatan, Pleasanton, CA) and 20-eV slit width. Images were acquired at ×61,000 nominal magnification, corresponding to a calibrated pixel size of 0.31 nm. Defocus values of −6 and −3 μm were used to image the virus-liposome complexes and virions, respectively. The total electron dose was 100 electrons/Å^2^ per tilt series. Images were down-sampled to 0.62 nm/pixel prior to reconstruction, and the IMOD package (28) was used to calculate three-dimensional tomograms. Prior to visualization and further processing, the tomograms were low-pass filtered to remove spatial frequencies higher than the first 0 in the contrast transfer function of the microscope (1/3.5 nm for tomograms taken at 6 μm underfocus; 1/2.5 nm for tomograms taken at 3 μm underfocus).

##### Subtomogram Averaging

To solve the structure of the UUKV glycoprotein hexamer, we extracted 96 virions from 9 tomograms for further processing in Jsubtomo (29, 30) following a gold-standard refinement protocol established earlier (31). Briefly, we first manually picked 54 glycoprotein capsomers from virion volumes and generated two independent template structures. These templates were used to detect and align all the capsomers in the virions for averaging. A frequency band between 1/3.5 and 1/18 nm was used in the final round of refinement. Capsomers with the highest cross-correlation and no overlaps to adjacent capsomers (3,008 capsomers) were included in the final average. In the average, the structure of the glycoprotein hexamer was resolved at 3.0-nm resolution as indicated by Fourier shell correlation at 0.143. For comparison to the structure of the RVFV hexameric capsomer, one capsomer was extracted from the published reconstruction (18) and filtered to 3.0-nm resolution.

## Results

### 

#### 

##### Uukuniemi Virus as a Model System to Study Phlebovirus Fusion

We chose UUKV as a model system to study phlebovirus fusion to target membranes, because UUKV has been a preferred model system to study phlebovirus entry earlier (11, 12). Unlike RVFV and severe fever with thrombocytopenia syndrome virus (SFTSV), which require high biosafety containment levels, UUKV is apathogenic and can be handled safely at a lower biosafety containment level.

It is widely assumed that the G_C_ proteins of phleboviruses share the class II fusion protein fold. We confirmed this assumption by bioinformatics analysis using the protein homology/analogy recognition engine (Phyre^2^), which allows detecting structural homologs even in the absence of notable sequence similarity (32). UUKV G_C_ protein sequence (UniProt: P09613) matched the best with RVFV G_C_ (UniProt: P03518; 26% identity) and 86% of the modeled UUKV G_C_ structure (residues 1–428 of total 495) could be modeled against the known RVFV G_C_ structure (Protein Data Bank code 4HJ1) with 100% confidence. This strongly supports the notion that the two proteins are homologous and share the same class II fusion protein-fold.

##### Electron Cryotomography Structure of Native UUKV

**FIGURE 1.**
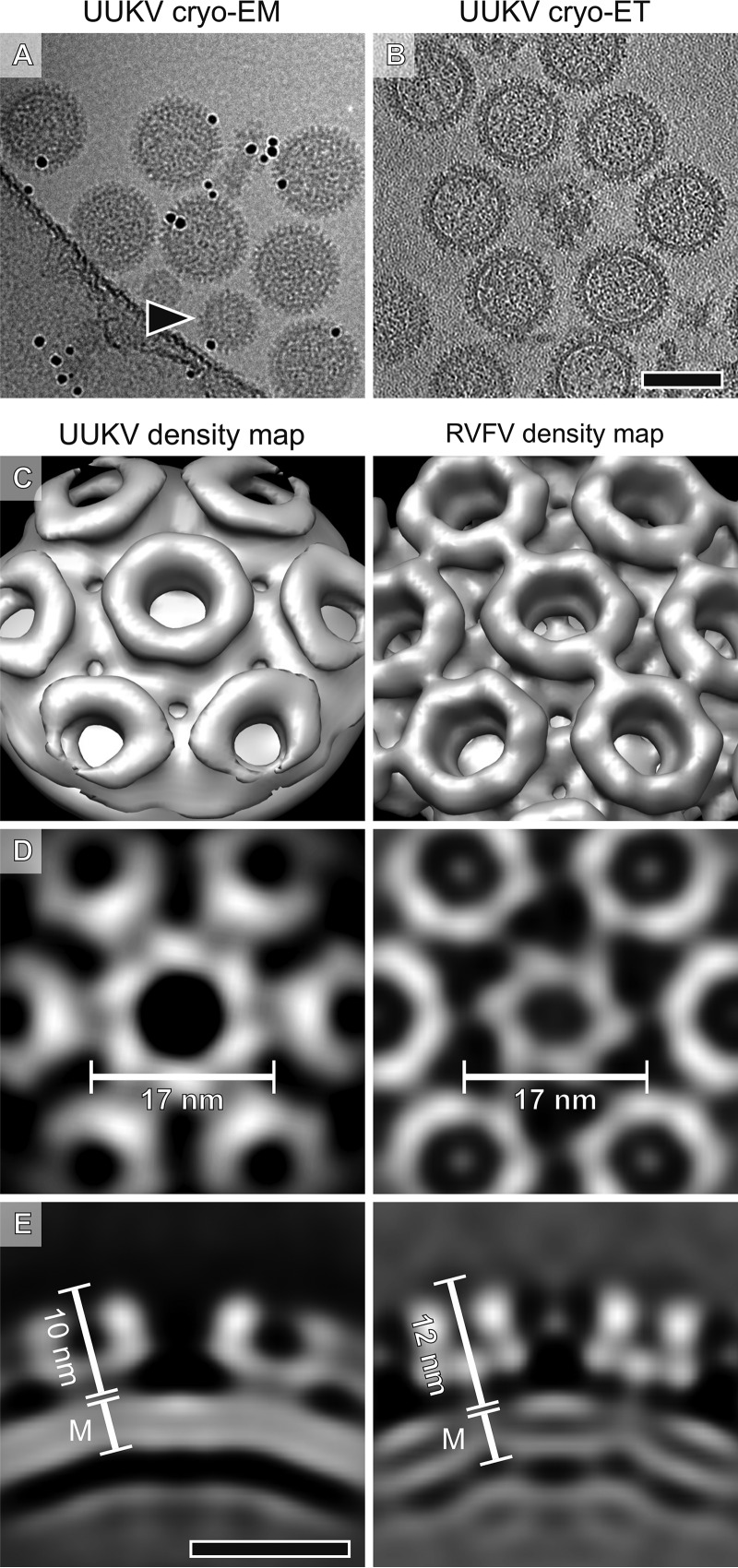
**Electron cryotomography and subtomogram averaging of UUKV virus.** *A,* a two-dimensional projection image of Uukuniemi (UUKV) virions at pH 7.5 collected at −6 μm defocus at 0° tilt. Gold nanoparticles (∼10 nm in diameter) have been added as fiducial markers to align images in tomographic tilt series. Virions were ∼110 nm in diameter and covered in glycoprotein capsomers. Smaller particles (∼50 nm) with virion-like morphology were occasionally observed (*arrowhead*). *B,* a 10-nm thick slice through a three-dimensional tomographic reconstruction of UUKV virions at pH 7.5 collected at −6 μm defocus. *Scale bar* = 100 nm for *A* and *B. C,* a surface rendering of the viral glycoprotein capsomers is shown for UUKV (*left*) and RVFV (*right*, EMDB-1550) solved using subtomogram averaging (this study, UUKV) and single particle icosahedral averaging (RVFV) (18). In both cases, one hexameric capsomer that is surrounded by six neighboring capsomers is shown. *D* and *E,* slices (6 nm thick) through the density maps are shown parallel (D) and orthogonal (*E*) to the viral membrane (*M*). Dimensions of the capsomers are indicated. *Scale bar* = 15 nm for *C–E*.

We studied the prefusion conformation of native UUKV particles at pH 7.5 by electron cryomicroscopy and tomography. Two-dimensional micrographs and three-dimensional tomographic reconstructions revealed particles that were nearly spherical in shape (Fig. 1, *A* and *B*). The particles displayed a continuous layer of glycoprotein capsomers on the surface. Subtomogram averaging of ∼3,000 glycoprotein capsomers from ∼100 virions at pH 7.5 yielded a structure of the native hexameric capsomer (width ∼17 nm, height ∼10 nm) at 3.0-nm resolution (Fig. 1, *C–E,* UUKV). This structure revealed a striking similarity to the cylindrically shaped hexameric capsomer of RVFV (18, 33) (Fig. 1, *C–E,* RVFV), but was different from the structure of the UUKV glycoprotein capsomers at neutral pH, fixed with glutaraldehyde, that we have reported earlier (34). The latter had a more pointed and elongated morphology (height ∼13 nm) and may correspond to a transient, extended conformation of the capsomers captured by the fixative. In conclusion, the native UUKV glycoproteins at pH 7.5 are organized as capsomers with a cylindrical shape comparable with that of the RVFV glycoprotein capsomers.

##### Fluorescent Membrane Labeling of UUKV

**FIGURE 2.**
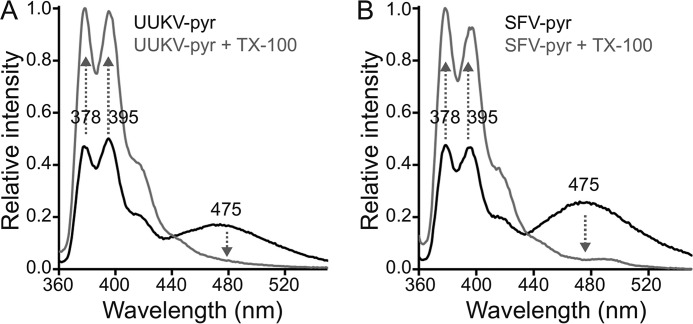
**Membrane labeling of UUKV and SFV with pyrene.** *A* and *B,* fluorescence emission spectra (excitation at 345 nm) are shown for pyrene-labeled Uukuniemi virus (*UUKV-pyr*) and Semliki Forest virus (*SFV-pyr*). Spectra were measured before (*black*) and after (*gray*) solubilizing the membrane with 0.2% (w/v) Triton X-100 (*TX-100*). Addition of Triton X-100 results in the solubilization of the membrane, and thus further dissociation of dimeric pyrene excimers. This is seen as increased emission (up arrows) at 378 and 395 nm corresponding to the pyrene monomers and as disappearance (down arrows) of the broad emission peak at 475 nm corresponding to the pyrene dimers.

Enveloped virus glycoprotein catalyzed fusion to target membranes has been studied earlier in virus-liposome fusion assays exploiting fluorescent membrane labeling (35, 36). Lipid mixing between virus and liposomes can be monitored by measuring changes in fluorescence upon dilution of pyrene-labeled phospholipids from metabolically labeled virions to unlabeled liposomes following a pH drop, as first described for vesicular stomatitis virus (35). Before conducting such experiments with UUKV, we confirmed the incorporation of fluorescent pyrene in sufficient amounts in the viral membrane to form excited dimers (excimers). Pyrene excimers emit a red-shifted fluorescence in the visible spectrum, as compared with monomeric pyrene, which emits in the UV range. As expected, pyrene excimers emitted light at 475 nm when excited at 345 nm and could be diluted almost completely to monomers not emitting at 475 nm, by addition of detergent (Fig. 2*A*).

In general, UUKV preparations have a rather high (>500) particle to PFU ratio (12). However, both labeled and unlabeled viruses had similar infectivity, as estimated by titration assays of viral preparations, in combination with SDS-PAGE analysis. The specific infectivity of UUKV and UUKV-pyr was 3.0 × 10^6^ ± 0.9 × 10^6^ FFU/μg (*n* = 2) and 7.4 × 10^6^ ± 2.4 × 10^6^ FFU/μg (*n* = 3), respectively (values are given as mean FFU/μg of viral glycoprotein ± S.E.). This showed that pyrene is incorporated into the virus particles at significant levels and without adverse effects on virus infectivity.

Similarly to UUKV-pyr, and to what has been published before (36), we also produced pyrene-labeled SFV (SFV-pyr) to use it as a control in our assays. SFV-pyr behaved similarly to UUKV-pyr (Fig. 2*B*).

##### Late Endosomal Phospholipid BMP Promotes Lipid Mixing

**FIGURE 3.**
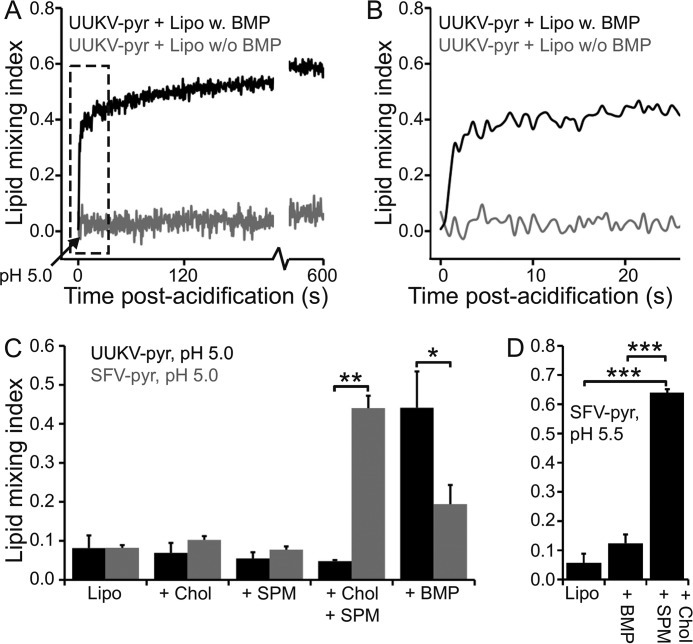
**BMP promotes lipid mixing between UUKV and liposomes.** *A* and *B,* pyrene-labeled Uukuniemi virus (UUKV-pyr) was mixed with either DOPC-DOPE (*Lipo w*/*o BMP*) or DOPC-DOPE-BMP (*Lipo w BMP*) liposomes and preincubated at 37 °C. After acidification to pH 5, lipid mixing was followed over time. In the presence of BMP, efficient lipid mixing was observed and, after 600 s, a plateau was reached at lipid mixing index of ∼0.6. No significant lipid mixing was observed in the absence of BMP. The first 25 s (indicated with a *box* in *A*) are shown in *B*. Most of the lipid mixing occurred in the first few seconds after acidification. *C,* quantification of UUKV-pyr and pyrene-labeled Semliki Forest virus (SFV-pyr; control) lipid mixing at 5 min post-acidification in the presence of liposomes. In addition to control DOPC-DOPE liposomes (*Lipo*), liposomes with added lipids were tested as indicated. As expected, SFV lipid mixing required both sphingomyelin (*SPM*) and cholesterol (*Chol*), whereas UUKV lipid mixing was only observed in the presence of BMP. *D,* quantification of SFV-pyr lipid mixing after 5 min at pH 5.5. No significant lipid mixing was observed in the presence of BMP and absence of SPM and Chol. Note that the *y* axis is on a different scale in *C* and *D. Error bars* represent 1 S.D. (*n* = 3). Statistical significance was determined using a Student's *t* test using significance levels: *, *p* ≤ 0.05; **, *p* ≤ 0.01; ***, *p* ≤ 0.001.

To study which target membrane phospholipid species have the potential to promote UUKV fusion, we used liposomes with different lipid compositions (Table 1). Because UUKV is believed to penetrate host cells from late endosomes (12) and may thus be expected to also undergo fusion in late endosomes, we hypothesized that not only late endosomal pH (below pH 6) but also the late endosomal lipid BMP may promote UUKV fusion. This lipid is highly abundant in the LE, constituting 15 mol % of the total LE phospholipids on the average and as high as 60 mol % in some LE fractions (37). Supporting our hypothesis, the presence of BMP promoted lipid mixing; at pH 5.0, UUKV-pyr could rapidly fuse to liposomes containing intermediate amounts of BMP (30 mol %; Fig. 3*A*). Most of the lipid mixing took place in the first few seconds after the pH drop (Fig. 3*B*) and reached maximal lipid mixing of 60% after 10 min (Fig. 3*A*).

**TABLE 1:** **Liposome preparations**

Lipids	Molar ratio
DOPC:DOPE	7.0:3.0
DOPC:DOPE:BMP	5.0:2.0:3.0
DOPC:DOPE:SPM:Chol	2.2:2.2:2.2:3.3
DOPC:DOPE:SPM	5.6:2.2:2.2
DOPC:DOPE:Chol	4.8:1.9:3.3
DOPC:DOPE:DOPS	5.0:2.0:3.0
DOPC:DOPE:DOPG	5.0:2.0:3.0
DOPC:DOPE:αPA	5.0:2.0:3.0
DOPC:DOPE:BMP:Chol	3.6:1.4:3.0:2.0
DOPC:DOPE:BMP:SPM:DOPS:PC-pyr:Chol	2.8:1.7:0.4:1.1:1.1:0.4: 2.5

To further validate this result, we compared the lipid requirements of UUKV-pyr to SFV-pyr, for which the conditions are well characterized (22, 25, 38, 39). At pH 5.0, no lipid mixing was observed between UUKV-pyr and the liposomes containing cholesterol and/or sphingomyelin but lacking BMP (Fig. 3*C*). Conversely, and consistent with earlier observations (22, 25), efficient SFV-pyr lipid mixing only occurred with liposomes containing both cholesterol and sphingomyelin (Fig. 3*C*). Unexpectedly, SFV-pyr also showed some lipid mixing to liposomes containing BMP in the absence of cholesterol and sphingomyelin (Fig. 3*C*). However, at pH 5.5, which is closer to the physiological SFV fusion threshold of pH ∼ 6 (9), lipid mixing to these liposomes was at background level (Fig. 3*D*), whereas UUKV-pyr still showed ∼50% lipid mixing (see Fig. 6*A*).

##### BMP Promotes UUKV-induced Cell-Cell Fusion at the Plasma Membrane

Alternative to virus-liposome fusion assays, virus fusion can be studied by forcing the fusion at the plasma membrane. In such a “fusion-from-without” assay, fusion events lead to syncytia formation, where two or more cells have fused together (40).

**FIGURE 4.**
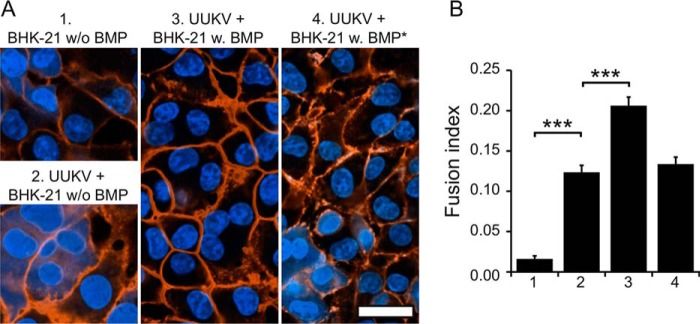
**BMP promotes UUKV-induced fusion of BHK-21 cells.** *A,* UUKV-induced cell-cell fusion visualized by fluorescence confocal microscopy. UUKV was added to BHK-21 cells, cell-cell fusion was induced by acidification to pH 5.0, and cell nuclei (*blue*) and plasma membrane (*orange*) were stained for counting the number of syncytia (*i.e.* cells with multiple nuclei). As a negative control, cells were first imaged without added virus or BMP (*1*). In the absence of added BMP, and in the presence of UUKV, some syncytia could be observed (*2*). The effect of BMP on syncytia formation was tested by adding BMP either before (*3*) or after (*4*) acidification. *Scale bar* = 25 μm. *B,* quantification of cell-cell fusion. Labels *1–4* are as in *A*. Note that BMP significantly increased syncytia formation only when added before acidification. *Error bars* represent the mean ± S.E. of the fusion index. Statistical significance was determined using a Student's *t* test using significance levels: *, *p* ≤ 0.05; **, *p* ≤ 0.01; ***, *p* ≤ 0.001.

To further study the role of the anionic phospholipid BMP in UUKV fusion, we measured fusion of UUKV to BHK-21 cells, either in the presence or absence of added BMP (Fig. 4*A*). We observed significantly increased levels of UUKV-induced cell-cell fusion at pH 5.0 when BMP was added to the cells before virus-binding and acidification/re-neutralization (Fig. 4*B*). However, if BMP was added after acidification/re-neutralization, fusion remained at the basal level (Fig. 4*B*). When only BMP, but no virus, was added, no significant fusion could be observed (not shown). As a control, the assay was repeated with SFV, which does not require BMP (22, 25). No increase in fusion was observed; in fact in the presence of BMP, significantly (*p* = 0.002) less fusion (0.25 ± 0.10) was observed than in its absence (0.33 ± 0.13).

Although the precise step at which BMP facilitates fusion remains unknown, these experiments further confirmed that BMP increases fusion between UUKV and target membranes. Interestingly, it has been demonstrated earlier in fusion-from-without assays that anionic lipids also promote fusion of dengue virus (20).

##### Viral Glycoproteins Are Required for Low pH-mediated Lipid Mixing to BMP-containing Liposomes

**FIGURE 5.**
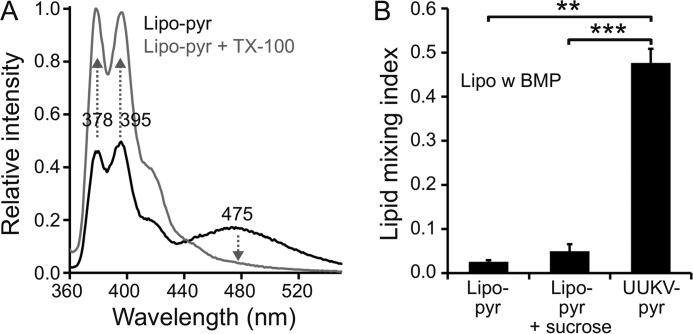
**UUKV glycoproteins are required for lipid mixing.** *A,* fluorescence emission spectrum of liposomes (*Lipo-pyr*) with a phospholipid composition similar to pyrene-labeled UUKV with and without added Triton X-100. *B,* quantification of lipid mixing between pyrene-labeled liposomes (*Lipo-pyr*) or Lipo-pyr with 2.5% (w/v) sucrose and unlabeled liposomes with BMP at 10 min post-acidification, pH 5.0. Pyrene-labeled Uukuniemi virus (*UUKV-pyr*) was added as a positive control for lipid mixing. No significant lipid mixing was observed in the case of labeled donor liposomes. *Error bars* represent 1 S.D. (*n* = 3). Statistical significance was determined using a Student's *t* test using significance levels: *, *p* ≤ 0.05; **, *p* ≤ 0.01; ***, *p* ≤ 0.001.

It has been previously reported that liposomes containing BMP can be intrinsically fusogenic at low pH (37). To exclude the possibility that the observed UUKV-pyr lipid mixing is passively caused by BMP in our assay, we produced pyrene-labeled donor liposomes (Lipo-pyr) containing similar monomer to excimer ratios than UUKV-pyr (Fig. 5*A*), and with practically the same phospholipid composition (Table 1) as reported for the UUKV virion earlier (41). We also included 25 mol % cholesterol in accordance with cholesterol levels found in the Golgi apparatus (42), the site of UUKV maturation (43). The fluorescent probe in the donor liposomes was a pyrene-labeled phospholipid analog 1-hexadecanoyl-2-(1-pyrenedecanoyl)-*sn*-glycero-3-phosphocholine (PC-pyr). Importantly, lipid mixing between pyrene-labeled donor liposomes and acceptor liposomes with BMP was at background level (Fig. 5*B*), showing that in our assay, glycoprotein-independent fusion played an insignificant role. Taken together, these results show that UUKV glycoproteins play an active role in promoting fusion to BMP-containing liposomes.

##### Lipid Mixing Requires Late Endosomal pH and Physiological Temperatures

**FIGURE 6.**
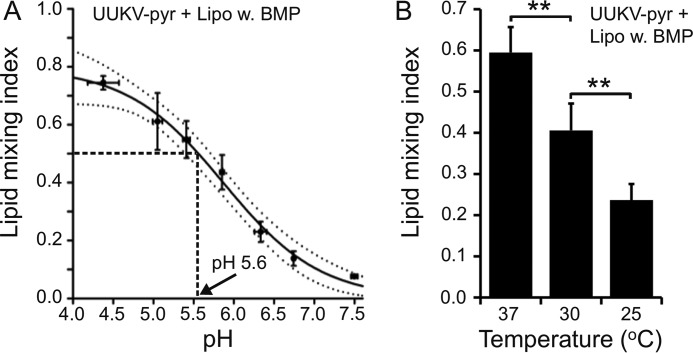
**UUKV lipid mixing with liposomes is pH and temperature sensitive.** *A,* lipid mixing of pyrene-labeled Uukuniemi virus (*UUKV-pyr*) with BMP containing liposomes was determined between pH 4.0 and 7.5 at 37 °C. For accurate pH measurements, pH was measured after acidification for each data point. *Error bars* represent 1 S.D. for both the pH (*horizontal bars*) and lipid mixing index (*vertical bars*; *n* = 3). A sigmoidal curve was fitted and the confidence of the fit is indicated with *dotted lines* (95% confidence level). Half-maximal lipid mixing was achieved at pH 5.6 (*dashed lines*). *B,* lipid mixing of UUKV-pyr with BMP-containing liposomes was determined at pH 5.0 at different temperatures. *Error bars* represent 1 S.D. (*n* = 4). Statistical significance was determined using a Student's *t* test using significance levels: *, *p* ≤ 0.05; **, *p* ≤ 0.01; ***, *p* ≤ 0.001.

The activation of the phlebovirus fusion machinery by acidic pH has been studied earlier by fusion-from-without assays. This has been demonstrated to occur at pH < 5.4 for UUKV particles (12) and at pH < 5.7 for RVFV (44) bound to the plasma membrane of BHK-21 cells. Consistent with this result, in our virus-liposomes fusion assay, we observed only minimal lipid mixing at neutral pH (<10%), whereas 50% efficiency was reached at pH 5.6 and the maximal efficiency below pH 5.0 (Fig. 6*A*). These observations are compatible with UUKV entry from LEs.

Lipid mixing efficiency was temperature dependent. Lipid mixing decreased rapidly at temperatures below 37 °C (Fig. 6*B*). For comparison, in an earlier UUKV fusion from without assay, fusion was reduced only by 30% at 20 °C when compared with the situation at 37 °C (12). The quantitative differences in the total extent of fusion, observed between our results with liposomes and the previously published results in the presence of cells, was probably due to using two different experimental systems. We conclude that UUKV lipid mixing to liposomes shows steep temperature dependence.

##### UUKV-induced Content Release from BMP-containing Liposomes at Low pH

The viral membrane fusion mechanism is thought to involve (i) a conformational change in the viral fusion proteins that is triggered by low pH and that leads to the insertion of the fusion peptides into the target membrane, (ii) further conformational changes bringing donor and acceptor membranes into close proximity, followed by mixing of the outer leaflets of the two membranes leading to hemifusion, (iii) progression from the hemifusion intermediate to fusion pore formation, leading to lipid mixing between the inner leaflets of the two membranes, and finally, (iv) fusion pore expansion, leading to content mixing between the two fused compartments (45–47).

Because partial lipid mixing already occurs in the hemifusion state, lipid mixing alone is not indicative of full fusion. To this end, we sought to measure content release from content-labeled liposomes in the presence of UUKV at low pH. We used liposomes that encapsulated a water-soluble fluorescent dye, sulforhodamine B (SRB), in self-quenching concentrations (48, 49). Leakage of SRB from the liposomes, either due to fusion pore opening and subsequent SRB release into the virus interior, or due to SRB leakage to the surrounding solution, leads to dilution of SRB. This in turn results in dequenching of fluorescence that can be measured (49). To allow measuring lipid mixing and content release in parallel, we used UUKV-pyr in these experiments.

**FIGURE 7.**
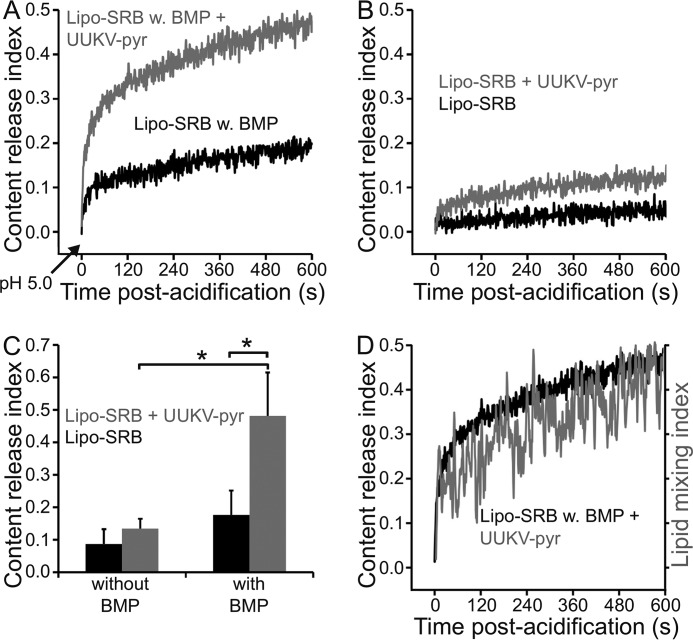
**UUKV induces content release from liposomes in the presence of BMP.** *A* and *B,* lipid mixing of pyrene-labeled Uukuniemi virus (*UUKV-pyr*) with DOPC-DOPE liposomes labeled with SRB (*Lipo-SRB*) was determined at pH 5.0, 37 °C. In the presence of BMP, a rapid UUKV-induced content release was observed as shown in *A*. In the absence of BMP, content release remained at background levels as shown in *B*. Notice that also in the absence of UUKV (*black trace*) some spontaneous release of SRB was observed. *C,* quantification of content release at 600 s post-acidification confirmed that UUKV-induced content release was significantly higher in the presence of BMP than in its absence. Furthermore, UUKV-induced content release was significantly higher than the spontaneous release of SRB dye from the liposomes. *Error bars* represent 1 S.D. (*n* = 3 for Lipo-SRB and *n* = 4 for Lipo-SRB with BMP). Statistical significance was determined using a Student's *t* test using significance levels: *, *p* ≤ 0.05; **, *p* ≤ 0.01; ***, *p* ≤ 0.001. *D,* simultaneous measurement of content release (*black trace*) and lipid mixing (*gray trace*) in the presence of UUKV-pyr and BMP containing liposomes labeled with SRB showed that content release starts concomitantly to lipid mixing and follows roughly the same kinetics.

Significant content release, as measured by SRB dequenching, occurred rapidly when UUKV was mixed with DOPC-DOPE-BMP-SRB liposomes (Fig. 7, *A* and *C*) at 37 °C and pH 5.0, but not with DOPC-DOPE-SRB liposomes (Fig. 7, *B* and *C*). Some dequenching occurred over time also in the absence of the virus, suggesting that some dye leaked out from the acidified liposomes spontaneously (Fig. 7, *A* and *B*). This is in contrast to earlier findings indicating that SRB-labeled DOPC liposomes were fully stable at pH 5.0 (49) and likely reflects the different lipid composition of the liposomes.

Content release was concomitant to lipid mixing, with no detectable delay (Fig. 7*D*). As the measured content release is a time average of several largely unsynchronized events, short delays in fusion pore opening cannot be detected with this assay. Single virus fusion assays are expected to yield more accurate information on the kinetics of hemifusion intermediate formation and fusion pore formation, as has been demonstrated for influenza virus earlier (50). Furthermore, we cannot exclude the possibility that some, if not all of the observed content release corresponds to “leaky fusion,” where soluble dye leaks out of the liposomes into the surrounding solution, instead of entering the viral interior through a fusion pore. Taken together, these data show a clear virus-induced target membrane destabilization in the presence of the phospholipid BMP, leading to liposome content release.

##### Anionic Phospholipids with Negative Spontaneous Curvatures Promote Fusion

**FIGURE 8.**
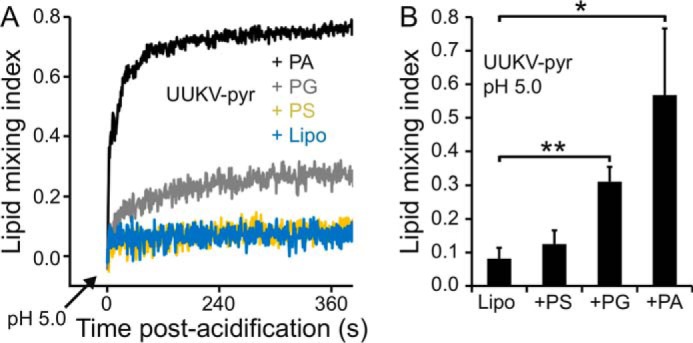
**UUKV lipid mixing with liposomes containing non-physiological anionic lipids.** *A,* pyrene-labeled Uukuniemi virus (*UUKV-pyr*) lipid mixing was measured with control DOPC-DOPE liposomes (*Lipo*) and with three other DOPC-DOPE liposomes containing additional phospholipids with negatively charged head groups, namely PG, PS, and PA. After acidification to pH 5.0, lipid mixing was followed over time. In the presence of PA and PG, efficient lipid mixing was observed. In the presence of PS, lipid mixing was at background level. *B,* quantification of UUKV-pyr lipid mixing at 5 min post-acidification in the presence of the same liposomes as in *A*. Liposomes with PG and PA showed significantly higher lipid mixing than the control liposomes (*Lipo*). *Error bars* represent 1 S.D. (*n* = 3). Statistical significance was determined using a Student's *t* test using significance levels: *, *p* ≤ 0.05; **, *p* ≤ 0.01; ***, *p* ≤ 0.001.

What properties of BMP facilitate UUKV fusion? As the BMP headgroup is negatively charged, we hypothesized that negative charge is required for lipid mixing and thus also for full fusion. To test this hypothesis, we included liposomes containing three other phospholipids with negatively charged headgroups, namely DOPG, PA, and DOPS, in our assays (Fig. 8*A*). One should note that none of these lipids are usually enriched in the inner membrane leaflets of late endosomes and thus are not expected to be physiologically relevant for UUKV fusion. Similarly to BMP, DOPG and PA facilitated efficient lipid mixing at pH 5.0. However, contrary to our hypothesis, no significant lipid mixing was observed in the presence of DOPS (Fig. 8*B*).

Which properties differentiate DOPS from the other anionic lipids tested? Unlike PA (51), PG (52), and BMP (37), which are thought to have negative spontaneous curvature at neutral pH, PS has been reported to have positive spontaneous curvature at neutral pH (53). Thus, negative spontaneous curvature might be one of the properties that promote UUKV lipid mixing. This hypothesis is in agreement with current membrane fusion models, which emphasize the role of phospholipids with negative spontaneous curvature in promoting lipid mixing (46, 54).

Interestingly, the spontaneous curvature of PS is known to change from positive to negative at pH 4.0 (53). Thus, if our hypothesis is correct and UUKV lipid mixing is promoted by both negative charge and negative curvature, also PS should promote fusion at pH 4.0. To test this, we measured UUKV lipid mixing at pH 4.0 to DOPC-DOPE-DOPS liposomes. In support of our hypothesis, significant lipid mixing (fusion index of 0.33 ± 0.03, compared with 0.09 ± 0.02 with DOPC-DOPE liposomes; *p* = 0.0007, *n* = 3) was observed. In addition, as the zwitterionic DOPE that has negative spontaneous curvature did not promote UUKV lipid mixing (see Fig. 3*C*, *Lipo*), curvature may not be the only factor promoting lipid mixing. Instead, our data suggests that both negative charge and negative spontaneous curvature are among the chemical and physical factors that promote UUKV fusion with lipid bilayers.

##### Electron Cryotomography of the UUKV-Liposome Fusion Complexes

**FIGURE 9.**
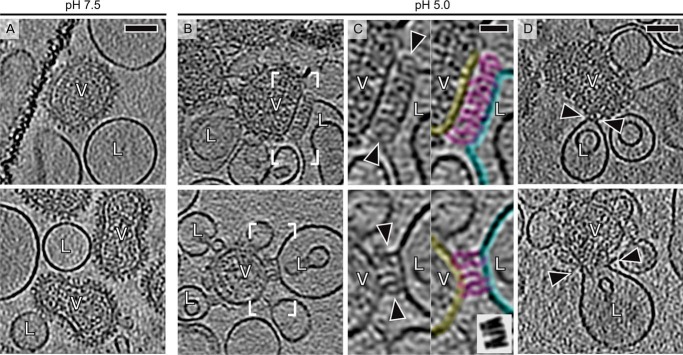
**Electron cryotomography of UUKV fusion.** *A* and *B*, shown are 10-nm thick tomographic slices of UUKV (V) with liposomes (*L*) at either pH 7.5 (control) or 5.0. Liposomes shown contained BMP and liposomes in the *lower panel* in *B* had also added cholesterol (see Table 1). Tomograms have been low-pass filtered to 6 nm^−1^ spatial frequency to emphasize the membranes and capsomers. *Scale bar* = 50 nm. *C,* a close-up of the areas indicated in *B* are shown. Regions with extended capsomer structures (*magenta*) connecting the virus membrane (*yellow*) to the liposome membrane (*cyan*) are indicated with *arrowheads* in the panel on the *left* and colored in the panel on the *right*. For size comparison, five G_C_ monomers in an extended conformation (Protein Data Bank code 4HJC) (14) were placed side by side in different orientations around the long axis and filtered to the same spatial frequency (*inset*). Note that such extended structures were also observed in the absence of BMP. *Scale bar* = 15 nm. *D,* a dimple structure at the virus-liposome contact site (*upper panel*) and a putative full fusion site where viral and liposome membranes have fully merged (*bottom panel*) are indicated with *arrowheads. Scale bar* = 50 nm.

Having found and characterized the minimal conditions for UUKV fusion with liposomes, we proceeded to study possible fusion intermediates by using electron cryotomography (Fig. 9). As expected, no UUKV-liposome fusion complexes were observed in the neutral pH control (Fig. 9*A*). However, UUKV mixed with BMP-containing liposomes at pH 5.0 revealed three different types of virus-liposome fusion complexes (Fig. 9, *B–D*). First, clusters of viral glycoprotein capsomers in an elongated conformation (membrane to membrane distance ∼18 nm) were observed to connect the viral membrane to the liposome membrane (Fig. 9, *B* and *C*). These elongated capsomers match the length of the RVFV G_C_ protein in the observed extended conformation (14) (Fig. 9*C*, *inset*) and were clearly different from capsomers at neutral pH (Figs. 1, *A* and *B,* and 9*A*) and capsomers in other areas of the virion (Fig. 9, *B–D*), which were ∼10 nm in length. This conformation was also observed in acidified liposomes that lacked BMP (not shown) suggesting that BMP is not required for inducing this conformation. Second, and similarly to what has been observed in influenza virus (49), UUKV readily induced high curvature regions, or “dimples,” on the liposome membrane at liposome-virus contact sites (Fig. 9*D*, *top*). Third, we also observed a putative fusion site where the viral membrane was continuous with the liposome membrane (Fig. 9*D*, *bottom*). As these observations provide snapshots of the fusion process, we cannot exclude the possibility that some observed structures, for example, capsomers in the elongated conformation, correspond to dead-end structures that would not culminate in fusion. Despite this limitation, our results provide direct evidence that UUKV undergoes fusion with BMP-containing membranes at pH 5.0. This is probably mediated by a conformational change in the viral glycoprotein capsomers taking place at virus membrane contact sites but further studies are required to verify this.

## Discussion

We established a minimal component virus-liposome model system for biophysical and structural characterization of phlebovirus fusion. Acidic pH was necessary but not sufficient for efficient UUKV fusion to liposomes; negatively charged phospholipids with a conical shape, notably BMP, were shown to facilitate efficient lipid mixing and content release.

Electron cryotomography and subtomogram averaging of UUKV glycoprotein capsomers revealed a structure similar to that of RVFV (17, 18). Furthermore, by using computational fold-recognition, we verified the assignment of UUKV G_C_ into the class II of viral fusion glycoproteins. These results suggest a high degree of structural conservation between the two phleboviral G_C_ glycoproteins.

It is widely accepted that acid-induced conformational changes in the class II viral fusion glycoprotein lead to the insertion of a hydrophobic fusion peptide in the target membrane via a transient extended conformation (47). Interestingly, electron cryotomography of UUKV-liposome mixtures acidified to pH 5.0 revealed elongated capsomer clusters bridging viral and liposome membranes. We attribute the appearance of these elongated capsomers to conformational changes in UUKV G_C_ fusion glycoprotein. These changes may correspond to either straightening of the G_C_ structure or to reorientation of G_C_ relative to the viral membrane. These elongated capsomers were limited to virus-target membrane contact sites. It is possible that in the parts of the virus that are not facing a target membrane, either no conformational changes take place, or the G_C_ glycoprotein folds back onto the virion surface to shield the hydrophobic fusion loop from the surrounding aqueous environment as much as possible. Similar elongated capsomer clusters have recently been observed in alphavirus-liposome complexes studied by electron cryomicroscopy (55), suggesting that the extended glycoprotein conformation is a general feature of class II fusion glycoproteins. It is tempting to speculate that the observed extended low pH conformation is analogous to the extended conformation observed in the crystallographic structure of the deglycosylated RVFV fusion glycoprotein G_C_ ectodomain (14) but this requires experimental verification.

What is the physiological relevance of the observed phospholipid and low pH dependence for UUKV fusion *in vivo*? A growing body of evidence suggests that not only pH, but also compartment-specific lipids and cholesterol concentration regulate endocytotic virus entry (20, 56, 57). The concentration of cholesterol and sphingomyelin decreases during the endocytic route, whereas the concentration of BMP drastically increases in the late endosomes (58, 59). Our findings are consistent with the known host cell penetration site of UUKV (*i.e.* late endosomes). As expected, cholesterol and sphingomyelin did not promote UUKV lipid mixing. On the other hand, BMP did promote efficient lipid mixing and also content release. Thus it is possible that BMP acts an entry cofactor, but this notion requires verification in further studies.

An interesting parallel is emerging between UUKV and dengue virus, an important late penetrating virus that also harbors a class II fusion glycoprotein. Similarly to UUKV, dengue has been reported to undergo fusion mainly in the LEs (60). In virus-liposome fusion assays, efficient lipid mixing has been shown to occur at pH 5.3 between dengue virus and either PC-PE-PI-BMP or PC-PE-PI-BMP-cholesterol liposomes, but not liposomes containing PC, PE, sphingomyelin, and cholesterol. The requirement for anionic lipids has been suggested to explain fusion in the LE (20). However, we also observed some differences in UUKV lipid mixing behavior, as compared with dengue. Levels of UUKV lipid mixing showed a clear discrimination between the species of negatively charged lipids tested. For example, phosphatidylserine did not allow lipid mixing at pH 5.0. This was in contrast to dengue virus, where lipid mixing levels to liposomes were statistically the same at pH 5.3, independently of whether PS, BMP, or PG was tested (20).

In conclusion, our results show that the anionic phospholipid BMP promotes phlebovirus membrane fusion in a virus-liposome model system. Further cell culture-based studies are required to assess the role of BMP during viral endocytosis. Furthermore, our work demonstrates that the UUKV fusion glycoprotein actively mediates low pH induced membrane fusion by undergoing a conformational change from a flat pre-fusion conformation to an elongated conformation attacking the target membrane. As viral membrane fusion is one of the major steps that could be targeted by antivirals to block virus entry, it will be of major interest to extend this study to other phleboviruses, many of which are important human and animal pathogens.

## Author Contributions

D. B. and J. T. H. designed the study, performed the experiments, analyzed the results, and wrote the paper. S. H. performed cell-cell fusion assays. A. C. performed electron cryotomography of native Uukuniemi virus. All authors reviewed the results and approved the final version of the manuscript.

## Footnotes

3 The abbreviations used are:

SFV: Semliki Forest virus
LE: late endosome
RVFV: Rift Valley fever virus
UUKV: Uukuniemi virus
BMP: bis(monoacylglycero)phosphate
BHK: baby hamster kidney
FFU: focus-forming unit
PFU: plaque-forming unit
DOPC: 1,2-dioleoyl-*sn*-glycero-3-phosphocholine
DOPE: 1,2-dioleoyl *sn*-glycero-3-phosphoethanolamine
Chol: cholesterol
DOPG: 1,2-dioleoyl-*sn*-glycero-3-phospho-(1′-rac-glycerol)
DOPS: 1,2-dioleoyl-*sn*-glycero-3-phospho-l-serine
PA: phosphatidic acid
PC-pyr: 1-hexadecanoyl-2-(1-pyrenedecanoyl)-*sn*-glycero-3-phosphocholine
SRB: sulforhodamine B
PI: phosphatidylinositol
PG: phosphatidylglycerol
PS: phosphatidylserine
SPM: sphingomyelin.
